# Link between bacterial communities and contrasted loads in ectoparasitic monogeneans from the external mucus of two wild sparid species (Teleostei)

**DOI:** 10.1186/s42523-024-00329-0

**Published:** 2024-07-30

**Authors:** Judith Revault, Yves Desdevises, Élodie Magnanou

**Affiliations:** Sorbonne Université, CNRS, Biologie Intégrative des organismes marins, BIOM, Observatoire Océanologique, Banyuls/Mer, F-66650 France

**Keywords:** External mucus, Microbiota, Sparidae, Monogenean, *Lamellodiscus*, Metabarcoding, Tripartite interaction

## Abstract

**Background:**

While teleost fishes represent two thirds of marine vertebrates, the role of their external microbiota in relationship with their environment remains poorly studied, especially in wild populations. Hence, the interaction of their microbiota with ectoparasites is largely unknown. Microbiota can act as a protective barrier against pathogens, and/or be involved in host recognition by parasites. Thus, host-parasite associations should now be considered as a tripartite interplay where the microbiota shapes the host phenotype and its relation to parasites. Monogeneans (Platyhelminthes) are direct life cycle ectoparasites commonly found on teleost skin and gills. The role of bacterial communities within skin and gill mucus which either pre-exist monogeneans infestation or follow it remain unclear. This is investigated in this study using the association between Sparidae (Teleostei) and their specific monogenean ectoparasites of the *Lamellodiscus* genus. We are exploring specificity mechanisms through the characterization of the external mucus microbiota of two wild sparid species using 16s rRNA amplicon sequencing. We investigated how these bacterial communities are related to constrated *Lamellodiscus* monogeneans parasitic load.

**Results:**

Our results revealed that the increase in *Lamellodiscus* load is linked to an increase in bacterial diversity in the skin mucus of *D. annularis* specimens. The date of capture of *D. annularis* individuals appears to influence the *Lamellodiscus* load. Correlations between the abundance of bacterial taxa and *Lamellodiscus* load were found in gill mucus of both species. Abundance of *Flavobacteriaceae* family was strongly correlated with the *Lamellodiscus* load in gill mucus of both species, as well as the potentially pathogenic bacterial genus *Tenacibaculum* in *D. annularis* gill mucus. Negative correlations were observed between *Lamellodiscus* load and the abundance in *Vibrionaceae* in gill mucus of *D. annularis*, and the abundance in *Fusobacteria* in gill mucus of *P. acarne* specimens, suggesting potential applications of these bacteria in mitigating parasitic infections in fish.

**Conclusions:**

Our findings highlight the dynamic nature of fish microbiota, in particular in relation with monogeneans infestations in two wild sparid species. More generally, this study emphasizes the links between hosts, bacterial communities and parasites, spanning from the dynamics of co-infection to the potential protective role of the host’s microbiota.

**Supplementary Information:**

The online version contains supplementary material available at 10.1186/s42523-024-00329-0.

## Introduction

Any organism is likely to host one or more parasitic species during its lifetime [1]. However, this does not mean that all hosts are equal face to parasitism: variability can be observed in the probability of infection (prevalence) and in the number of parasites within each host (parasitic load). Recently, among the avenues explored to explain these variations in host specificity, an increasing number of studies [2–4] suggest a role of the microbiota in host-parasite interactions. Indeed, several studies have shown that parasitized hosts possess a specific microbiota that differs from non-parasitized hosts, emphasizing the putative involvement of the microbiota in host-parasite interactions [5–9]. However, it remains unclear whether some of these differences in host bacterial communities are a cause or a consequence of parasitic infections.

Certain bacteria have been shown to protect the host against parasitic infections. This is the case, for example, in bumblebees, where several studies have shown a protective effect of the gut microbiota against a trypanosomatid parasite [10, 11]. Conversely, bacteria pre-existing the infection could favor the establishment and survival of parasite by creating favorable conditions. In 2010, Hayes et al. [12] showed that reducing the number of bacteria in the large intestine of mice significantly reduced the number of hatched nematode eggs. Other authors have shown that the microbiota can be involved in the attraction of parasites toward their host via chemical cues. This has for example been shown for mosquitos that are attracted by the skin microbiota of certain human hosts [13]. Another recent study has shown that cane toad skin secretions (i.e., substances produced by amphibians and their microbiota) attract lungworm larvae and enhance their infection success [14]. Interestingly, these authors showed that, depending on the geographical area, these same skin secretions could also reduce the longevity and infection success of the parasite larvae.

Differences in bacterial communities also seems to be the result of parasite infestation. In the case of helminth infestation, it has been shown that parasites induce a change in the composition of the microbiota [15], reducing airway inflammation in mice to prevent their expulsion, and that this mechanism is suppressed in hosts that have received antibiotics [16, 17]. A recent study suggest that infection by a crustacean ectoparasite (*Tracheliastes polycolpus*) induced a shift of the fin microbiota in a freshwater fish (*Leuciscus burdigalensis*), and this shift was restricted to the fin where the parasite anchored [18]. Thus, microbiota has been shown to reflect the infection status of the host [3].

Deciphering the role of the microbiota in parasitic infection can be pertinent in fields such as aquaculture, where parasites are responsible for considerable annual economic losses [19]. This is the case for monogeneans [20], ectoparasites abundant on teleost skin and gills. Teleost skin and gills are covered with a mucus colonized by stable communities of microorganisms that form the external microbiota. Several studies have shown that monogeneans detect their hosts via cues emitted by fish mucus [21–24] such as mucosal macromolecules (IgM) [25] and glycoproteins [26, 27]. Recent work suggests that the molecules involved in the monogeneans’ host recognition system are produced by the host microbiota [28]. Among the many roles assumed by the microbiota for its host fish [29–31], its link with pathogens and parasites is not well understood. One of the difficulties is to disentangle causes from consequences, particularly in wild populations where the microbiota is under the influence of numerous biotic and abiotic factors [32–35].

In the Mediterranean Sea, the association between the Sparidae (Perciformes) fish family and their specific monogeneans belonging to the *Lamellodiscus* genus is a relevant model to study fish-monogenean association. *Lamellodiscus* monogeneans are ectoparasites with a direct life cycle often abundant on the skin and gills of sparids. The establishment of monogeneans on their host is regulated at multiple levels, involving two critical stages: when the swimming larvae (oncomiracidia) seek out, reach and attach on fish skin, and when the larvae move, attach and establish in the gills. Previous studies have shown that certain highly specific monogenean larvae are able to parasitize fish species not recorded as their host species [24, 36]. Ohhashi and coll. [24] have proposed that these monogenean larvae could only survive for a few days and finally detach from their non-specific host, being unable to mature to the adult stage in the gills.

The aim of this study was to characterize the differences in bacterial communities patterns in the external mucus (skin and gill) of two Mediterranean sparid species (*Diplodus annularis* and *Pagellus acarne*) with contrasting *Lamellodiscus* monogeneans parasitic loads. *Lamellodiscus* abundance and diversity were assessed to investigate their effect on the structure and diversity of bacterial communities in each individual’s external microbiota (i.e., the skin and gill mucus). The effect of sampling conditions (i.e., location and date of capture) on these two microbiota was also investigated. We focused our analysis on gill microbiota, where the parasites settle definitively as adults, to look for correlations between the abundance of a given bacterial taxa and *Lamellodiscus* abundance in both sparid species.

## Materials and methods

### Fish sampling, DNA extraction, 16s RNA sequencing and processing

Details of the protocols for fish sampling, DNA extraction, 16S rRNA sequencing and sequence processing protocols can be found in [35]. Briefly, fish sampling of *D. annularis* individuals was conducted in June and July 2021 in the bay of Banyuls-sur-Mer and fish sampling of *P. acarne* individuals was conducted in June, July 2021 and March 2022 in both bays of Banyuls-sur-Mer and Argelès-sur-Mer (northwestern Mediterranean Sea, France) (Supplementary table S1). All fish were collected from a net with gloves and immediately taken to the laboratory for dissection; skin mucus and gill mucus were collected with sterile spatula and immediately placed in sterile tubes and either frozen at -80 °C until DNA extraction. For convenience, we will refer to gills and skin in this study as “tissues”. A total of 28 fish individuals were sampled for their skin and gill mucus, all belonging to two sparid species: 12 individuals identified as *Diplodus annularis* and 16 individuals as *Pagellus acarne* (Table 1). For each fish sampling done in Banyuls-sur-Mer (4 sampling dates), two liters of seawater were collected in a sterile container and filtered through a 0.2 μm nitrocellulose filter which was stored at -80 °C prior to DNA extraction.

DNA was extracted by using the Quick-DNA Fecal/Soil Microbe MiniPrep Kit (Zymo Research, Orange, California). PCR amplification was carried out in triplicate and performed using primers targeting the hypervariable V3-V4 region of the 16S rRNA gene: 341 F (5’CCTACGGGNGGCWGCAG-3′) and 805R (5′-GACTACHVGGGTATCTAATCC-3′) [37, 38]. A second PCR was performed to add barcodes to each amplified sample and the Illumina adapters. The concentration of all PCR products was normalized with a 96 well SequalPrep Normalization Plate (Thermofisher, France). Amplicons were sequenced using Illumina 2 × 300 bp MiSeq sequencing (FASTERIS SA, Switzerland).

The analysis of the raw sequences was done using the QIIME2 software and the standard pipeline of DADA2 [39–41]. Raw reads were demultiplexed, quality checked and trimmed to remove primer regions, paired ends were assembled, chimeric sequences were discarded, and reads were denoised. DADA2 generate a list of Amplicon Sequence Variants (ASVs). Sequences were aligned against the SILVA 138 reference database distributed by the Silva project [42, 43]. The MAFFT program was used to align the sequences and FastTree to build a phylogenetic tree. Based on the classification, ASVs matching “Archaea”, “Eukaryota” and “Unassigned” or represented by a single sequence in all samples were removed.

**Table 1:** Total number of fish samples used in this study

	Gill mucus				Skin mucus				Total
	LP	HP	Total		LP	HP	Total		
*Diplodus annularis*	6	6	12		6	6	12		24
*Pagellus acarne*	8	8	16		8	8	16		32
Water	-	-	-		-	-	-		4

### Characterization of gill parasites

To characterize *Lamellodiscus* species diversity and abundance, *Lamellodiscus* individuals were recovered from three outermost gill arches for each fish individual and counted under a dissecting microscope. In this study, we will consider the total *Lamellodiscus* load without considering their species affiliation. Based on our previous results [35, 44] we determined that the mean intensity of *Lamellodiscus* in *D. annularis* specimens caught during the same season as ours was 13 *Lamellodiscus* per 3 gill arches. In *P. acarne* specimens caught during same seasons than ours, the mean intensity of *Lamellodiscus* was 21 per 3 gill arches. Consequently, for downstream analyses, individuals of each fish species were grouped into two categories: those with *Lamellodiscus* spp. intensity below the previously reported mean in the three dissected gill arches were classified as ‘lightly parasitized’ (LP); those with higher *Lamellodiscus* spp. mean intensity were classified as ‘heavily parasitized’ (HP). For simplicity, we will use the terms “heavily” or “lightly” parasitized to refer specifically to the parasite loads in *Lamellodiscus* spp.

### Data analyses

The influence of location and date of capture on *Lamellodiscus* abundance in the fish was assessed using a Mann-Whitney-Wilcoxon rank sum test in R, supported by permutation tests for robustness. α-diversity measures were estimated with Faith’s phylogenetic and Shannon indexes using the dataset normalized to the total number of sequences per samples and the function *transform_sample_count* in the *phyloseq* R package [45]. We performed one-way ANOVA (or non-parametric Kruskal-Wallis tests when data were not normally distributed) to compare alpha diversity between tissues (i.e., skin mucus, gill mucus and water) and species. When the ANOVA (or Kruskal-Wallis test) rejected the null hypothesis, we computed pairwise comparisons between group levels using Tukey post hoc tests (or post hoc Conover-Iman (CI) test) with Benjamini-Hochberg correction to detect significant differences between groups. Linear models (LM) or generalized linear model (GLM) (depending on the normal distribution of the data) were performed for each species to identify which fish-related factors (i.e. tissue, parasitism) or environment-related factors (collection date and capture location for *P. acarne* samples) influence fish bacterial diversity (*lme4* package : lm/glm/glm.nb(Diversity ~ Tissue * Parasitism * Collection date * Capture location). For modeling Faith’s phylogenetic diversity obtained from *D. annularis* samples, a negative binomial regression was used (as the variance is larger than the mean). A GLM with Gamma distribution was used for Shannon diversity in *P. acarne* samples. Correlations between *Lamellodiscus* parasitic load (i.e., total abundance) and gill mucus microbiota diversity (Faith’s and Shannon index) were computed and their significance assessed using Pearson’s correlation tests. Finally, the number of shared ASVs among gill mucus microbiota of LP and HP individuals from both fish species and surrounding water was calculated and represented using a Venn diagram [46] using a rarefied dataset (rarefaction performed to the minimum library size of *D. annularis* samples, 5691 reads).

Principal Coordinates Analysis (PCoA) using both Bray-Curtis, based on ASVs’ abundance, and weighted Unifrac distance, which takes into account both the ASVs’ abundance and their phylogenetic relationships, was used to assess the differences between the microbiota of the different fish species. Firstly, permutational multivariate analysis of variance (PERMANOVA (function *adonis*, *vegan* R package) and pairwise comparisons for Bray-Curtis and Weighted Unifrac indices (10,000 permutations) were used to to test for differences of PCoA groups between tissues (i.e., gill mucus, skin mucus and water) and species. For each tissue and species, we have performed additional multifactorial PERMANOVAs and used the *r*^2^ value from PERMANOVA to estimate the relative effect size (% of variation explained) of factors tested (date of capture, capture location, level of parasitism, and the interactions date of capture × capture location, date of capture × parasitism level, and capture location × parasitism level) on skin and gill microbiota of both species. Given that bacteria from the surrounding water could appear as transient bacteria on gill and skin mucus of fish, these analyses were also performed without considering sequences retrieved from water samples in *D. annularis* samples, for which surrounding water were available at each sampling date.

To assess how each bacterial taxon contributed to the dissimilarity between HP and LP gill or skin mucus bacterial communities, we performed a Linear discriminant analysis Effect Size (LEfSe) [47]. LEfSe provides Linear Discriminant Analysis (LDA) scores for the bacteria taxa contributing the most to the differences between bacterial communities. We calculated relative abundances (i.e., total sum scaling) of bacterial taxa (i.e., phyla, class, order, family and genus) showing a significant contribution to the dissimilarity between HP and LP bacterial communities.

Spearman’s rank correlation was used to investigate the putative link between the abundance of *Lamellodiscus* spp. and the composition of gill and skin bacterial communities at family and genus taxonomic level. A correlation between the abundance of a *Lamellodiscus* spp. and the abundance of a bacterial taxa was considered to be significant when *p*-*value* < 0.05. Only taxa with abundance > 5% and present in at least 2 samples were considered.

## Results

For this study, 12 individuals belonging to *Diplodus annularis* species were sampled between June and July 2021. Between 0 and 90 *Lamellodiscus* spp. were found in the 3 dissected gill arches depending on the fish individual; 6 were classified as “lightly parasitized” (LP) and the other 6 were classified as “heavily parasitized” (HP). We also collected 16 *Pagellus acarne* individuals between June 2021 and March 2022. We found between 0 and 189 *Lamellodiscus* individuals in the 3 dissected gill arches, 8 fish individuals were classified as LP and the 8 remaining as HP (Supplementary Table S1). In total, 511 721 sequences assigned to bacteria binned into 4299 ASVs were retrieved from 56 fish samples and 4 water samples.

### General patterns of bacterial composition

**Fig. 1:**
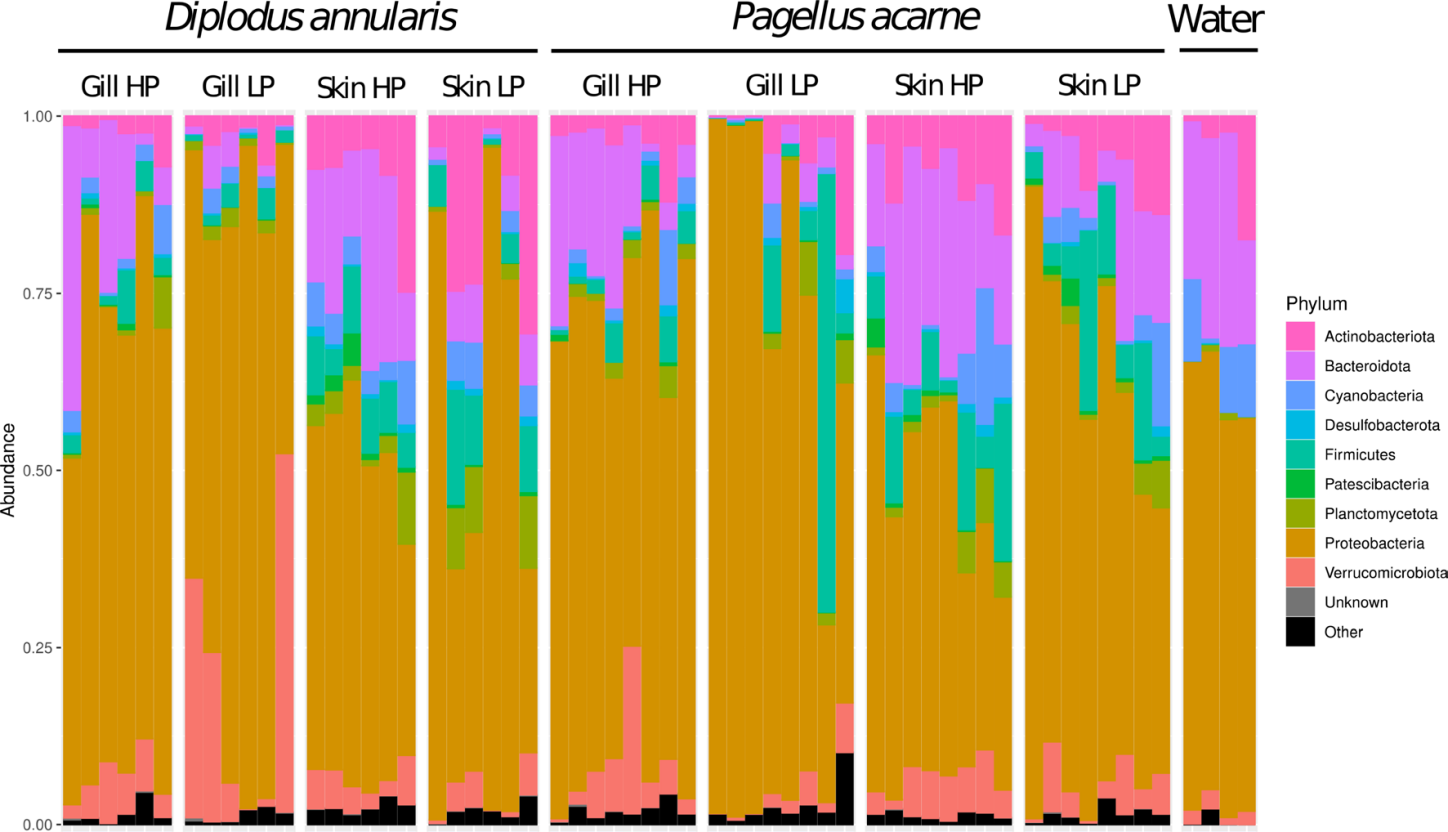
Relative abundance of bacterial phyla within lightly parasitized (LP) and heavily parasitized (HP) gill mucus or skin mucus of *Diplodus annularis* and *Pagellus acarne*

For the two fish species, the predominant bacterial phylum in gill and skin microbiota was *Proteobacteria* (67.74 ± 14.48% in relative abundance (i.e., total sum scaling method) in gill of *D. annularis*, 51.93 ± 22.39% for skin of *D. annularis*, 69.05 ± 20.74% for gills of *P. acarne* and 51.07 ± 16.92% for skin of *P. acarne*) (Fig. 1; Table 2). In *D. annularis*, the second most abundant phylum for gills was *Verrumicrobiota* (12.23 ± 15.70%) and *Actinobacteria* (12.53 ± 10.23%) for skin (Fig. 1; Table 2). *Bacteroidota* was the third most abundant phylum for both tissue in this species (9.15 ± 12.26% for gills and 12.14 ± 9.58% for skin) (Fig. 1; Table 2). In *P. acarne*, *Bacteroidota* was the second most abundant phylum found on the gills (8.30 ± 8.93%) and the skin (16.76 ± 9.39%) and *Firmicutes* the third most abundant in both tissues (6.85 ± 15.02% in gill and 9.28 ± 7.48% in skin) (Fig. 1; Table 2).

Figure 1: Relative abundance of bacterial phyla within lightly parasitized (LP) and heavily parasitized (HP) gill mucus or skin mucus of *Diplodus annularis* and *Pagellus acarne*.

**Table 2:** Relative abundance (percentage) of the 7 more represented bacterial phyla in *Diplodus Annularis*, *Pagellus acarne* and water microbiota (%)

	*Diplodus annularis*			*Pagellus acarne*			
	**Gill**	**Skin**		**Gill**	**Skin**		**Water**
Actinobacteriota	2.70	12.53		4.18	7.76		5.80
Bacteroidota	9.15	12.14		8.30	16.76		23.99
Cyanobacteria	2.07	4.17		1.83	4.59		8.04
Firmicutes	2.59	7.30		6.85	9.28		0.057
Planctomycetota	1.61	4.45		2.11	2.70		0.59
Proteobacteria	67.74	51.93		69.05	51.07		59.04
Verrumicrobiota	12.23	3.56		4.25	4.85		1.82

### Bacterial diversity of gill mucus and skin mucus

Two alpha diversity metrics were used to measure diversity within communities: the Faith’s phylogenetic index (phylogenetic richness) and the Shannon diversity index (taxonomic richness and evenness). When comparing diversity metrics, we found significant differences between the bacterial community of the *P. acarne* skin mucus, gill mucus and the surrounding water with both statistical tests (ANOVA; KW test, *p* < 0.05). This results from a highest bacterial diversity in skin mucus (Supplementary figure S1) but for both alpha diversity metrics, there was no significant differences in diversity between water and gill mucus (ANOVA; KW test, *p* < 0.05; CI tests, *p* > alpha/2 = 0.025, Post hoc Tukey *p* < 0.05) (Supplementary figure S1). In *D. annularis*, significant differences between bacterial communities in skin mucus, gill mucus and water were found only when considering the Faith index (KW test, *p* < 0.05) also explained by higher bacterial diversity in skin mucus (Supplementary figure S1).

**Fig. 2:**
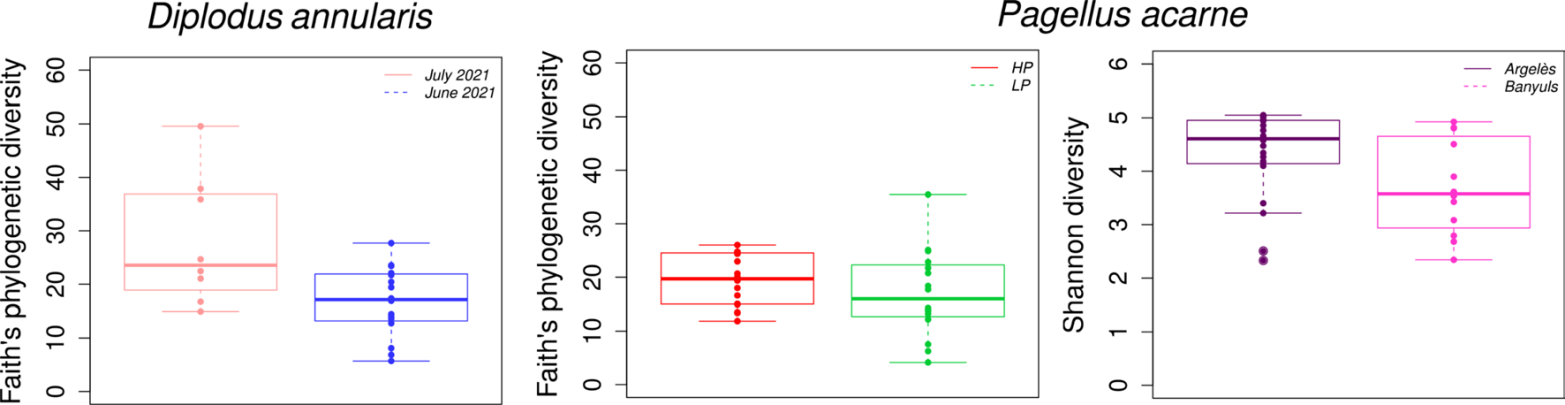
Faith’s phylogenetic or Shannon diversity index for date of collection of *D. annularis* (left); for lightly parasitized (LP, green) and heavily parasitized (HP, red) of *P. acarne* (center); for capture location of *P. acarne* (right)

When considering only fish samples, the LM and GLM results showed that, for both species, tissue (i.e., gill mucus and skin mucus) significantly influences Shannon diversity (Supplementary Table S2). HP and LP *P. acarne* bacterial communities were significantly different from each other when considering Faith’s index (LM, *p* < 0.05) with a higher bacterial diversity in HP individuals (Fig. 2). For *D. annularis*, collection date significantly influenced diversity when considering the Faith index (GLM, *p* < 0.05), with fish caught in July 2021 displaying a greater bacterial diversity (Fig. 2). In both species, no significant interaction between tissues and parasitism (i.e., HP vs LP) or collection date was found, regardless the diversity index considered (LM or GLM, *p* > 0.05). For *P. acarne*, collection site significantly influenced Shannon diversity (GLM, *p* < 0.05) and a significant interaction between tissue and collection site was also observed (GLM, *p* < 0.05), meaning that the collection site impacts differently each external fish tissue. In addition, the date of collection seems to have an influence on the parasitic load in *D. annularis* individuals (Mann-Whitney-Wilcoxon rank sum test, *p* < 0.01). On the contrary, place and date of capture did not significantly influence *Lamellodiscus* abundance (Mann-Whitney-Wilcoxon rank sum test, *p* = 0.11, *p* = 0.83 respectively) in *P. acarne* individuals.

### Factors explaining the dissimilarity of bacterial communities

To determine the factors explaining the variability between and within skin mucus, gill mucus and water microbiota, we used the Bray-Curtis dissimilarity index (BC), and the weighted Unifrac distance (WU). Principal coordinate analysis (PCoA) was used to plot both BC and WU distances.

First, significant differences between bacterial communities from gill mucus, skin mucus and surrounding water were obtained when considering all samples together (PERMANOVA on BC and WU distances, *p* < 0.05, R^2^ = 0.10 and R^2^ = 0.15 respectively). An effect of host species within gill and skin mucus on bacterial communities based on BC dissimilarities can be observed (PERMANOVA on BC distances, *p* < 0.01, 0.07 < R²<0.09) (Table 3).

**Table 3:** Results of multifactorial PERMANOVAs on factors explaining the variability in composition of bacterial communities. Significant *p-*values are highlighted in bold (PERMANOVA, *p* < 0.05)

		Gill mucus						Skin mucus				
		Bray-Curtis			W unifrac			Bray-Curtis			W unifrac	
		*p*-value	*R* ^2^		*p*-value	*R* ^2^		*p*-value	*R* ^2^		*p*-value	*R* ^2^
Both species	Species	**< 0.001**	0.07		0.75	-		**0.003**	0.09		0.2	-
*Diplodus annularis*	Collection date	0.09	-		0.07	-		**0.038**	0.16		0.7	-
	HP vs LP	**< 0.001**	0.16		**0.004**	0.41		**0.002**	0.24		**0.002**	0.68
*Pagellus acarne*	Collection date	**0.001**	0.19		**0.001**	0.71		**< 0.001**	0.26		0.7	-
	Sampling location	**< 0.001**	0.11		0.2	-		**< 0.001**	0.2		**0.02**	0.37
	HP vs LP	0.191	-		**0.02**	0.12		0.6	-		0.09	-
	Collection date x Sampling location	**< 0.001**	0.09		0.3	-		**0.002**	0.11		0.03	-
	Collection date x HP vs LP	0.5	-		0.02	-		0.87	-		0.06	-
	Sampling location x HP vs LP	**0.01**	**0.07**		0.08	-		0.88	-		0.01	-

For each species and each tissue, additional PERMANOVAs were performed to identify factors that influence the composition of the external microbiota. Due to the relatively close sampling of *Diplodus annularis* individuals over time, no significant effect of collection date on the variability of gill mucus bacterial communities was found based on BC and WU dissimilarities (PERMANOVA, *p* > 0.05). However, a significant effect of collection date was found on skin mucus based on BC dissimilarities (PERMANOVA, *p* < 0.05, R^2^ = 0.16) (Table 3). Significant differences between bacterial communities from LP and HP *D. annularis* gill and skin mucus were found (PERMANOVA on BC and WU distances, *p* < 0.05; for BC distances R²=0.16, R²=0.24 respectively; for WU distances R²=0.41, R²=0.68) (Table 3, Supplementary figure S2). The same significant results were obtained without considering sequences from water samples in *D. annularis* samples, with a small decrease in influence of the level of parasitism on gill and skin mucus microbiota dissimilarities (PERMANOVA on BC and WU distances, *p* < 0.05, R^2^ = 0.15 and R^2^ = 0.34 respectively for HP and LP gill and R^2^ = 0.21 et R^2^ = 0.56 for skin mucus).

In *P. acarne*, sampling conditions had a significant effect on variability of bacterial communities in gill and skin mucus when considering BC dissimilarities. If we consider the differences of diversity between July 2021 and March 2022 (as a single individual was captured in June 2021), sampling date seems to have a stronger effect (PERMANOVA, *p* < 0.001, R²=0.19, R²=0.26 respectively) compared to the sampling location (PERMANOVA, *p* < 0.001, R²=0.11, R²=0.2) on gill and skin mucus bacterial communities (Table 3). A significant effect of the interaction between collection date and sampling location was found for gill and skin microbiota of *P. acarne* individuals (PERMANOVA, *p* < 0.05, R^2^ = 0.09 and R^2^ = 0.11 respectively) and a small effect of the interaction between sampling location and parasitism (i.e., LP or HP) for gill microbiota considering BC dissimilarities (PERMANOVA, *p* < 0.05, R^2^ = 0.07) (Table 3). When phylogenetic relationships between ASVs were taken into account (WU dissimilarities), no significant differences were found in the skin microbiota of *P. acarne* fish over time (*p* > 0.05) in contrast to the gills where sampling date seemed to have a strong significant effect on microbiota (*p* < 0.05, R²=0.71).

Moreover, *P. acarne* individuals captured at both sites harbor a closely related gill microbiota (PERMANOVA, WU dissimilarities *p* > 0.05) but a significative effect of the location was found on skin microbiota (*p* < 0.05, R²=0.37) (Table 3). In *P. acarne*, parasitism (i.e., LP or HP) does not seem to explain the dissimilarity of bacterial communities from skin mucus (PERMANOVA on BC and WU distances, *p* > 0.05). However, a significant difference was observed in gill microbiota only when WU distances were considered (PERMANOVA, *p* < 0.05, R²=0.12) (Table 3).

**Fig. 3:**
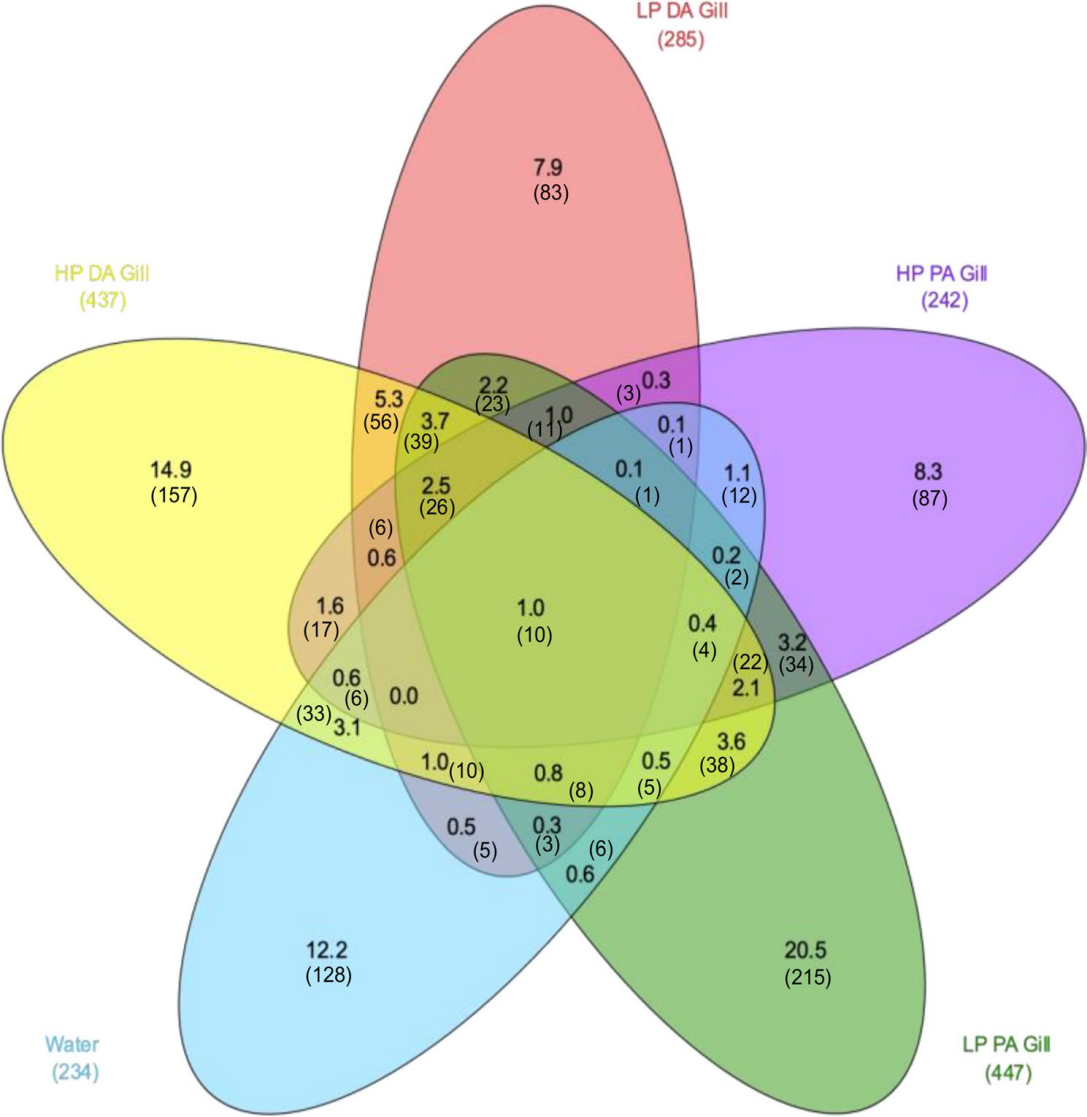
Venn diagram representing representing percentage of shared ASVs between lightly parasitized (LP) *P. acarne* gill mucus (green), heavily parasitized (HP) *P. acarne* gill mucus (purple), LP *D. annularis* gill mucus (green), HP *D. annularis* gill mucus (yellow) and water (blue). PA = *P. acarne*, DA = *D. annularis*. Based on 0.005% abundance cutoff

Gills of HP or LP *D. annularis* and HP or LP *P. acarne* harbor specific bacterial taxa (14.9%, 7.9%, 8.3%, 20.5% of ASVs respectively) (Fig. 3). These four compartments shared 2.5% of ASVs, mainly composed of *Proteobacteria* (*Alpha* and *Gammaproteobacteria*), followed by *Bacteroidota* as many *Firmicutes* as *Cyanobacteria*. The greatest compositional similarity was observed between LP and HP *D. annularis* (14.9%). It can be noted that LP individuals of both species share 118 ASVs (12.4%), of which 23 are shared solely between them, while HP individuals of both species share 91 ASVs (8.8%), of which 17 are shared solely between them (Fig. 3). 12.2% of ASVs are found only in water samples (corresponding to 55% of total ASVs retrieved from water samples) and are mainly composed by *Proteobacteria (Alpha* and *Gammaproteobacteria*) and *Bacteroidota* (Fig. 3).

### Correlations between bacterial communities and parasitic load in gills

The microbiota diversity assessed using Faith phylogenetic index in the skin mucus of *D. annularis* was positively correlated with *Lamellodiscus* load (Pearson correlation test, *p* < 0.05, *R* = 0.885) (Supplementary figure S3). However, at gill level, no correlation was found between changes in parasite load and microbiota diversity for *D. annularis* or *P. acarne* (*p* > 0.05).

**Fig. 4:**
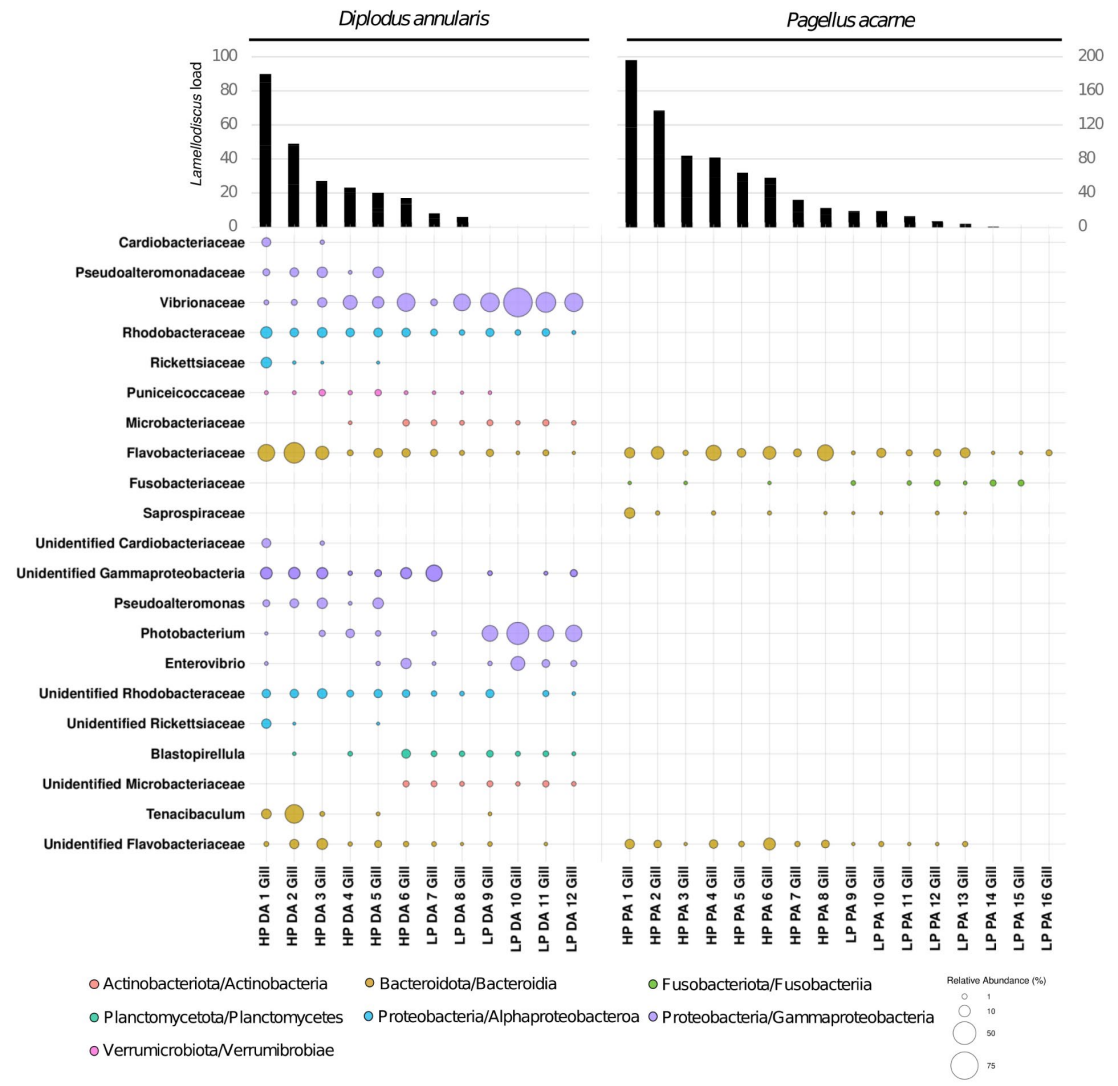
Significant correlations (Spearman rank correlation) between each taxonomic rank and parasitic load in gills. Only taxa with abundance > 5% and present in at least 2 samples are shown. DA, *Diplodus annularis*; PA, *Pagellus acarne.* LP, lightly parasitized; HP, heavily parasitized

**Fig. 5:**
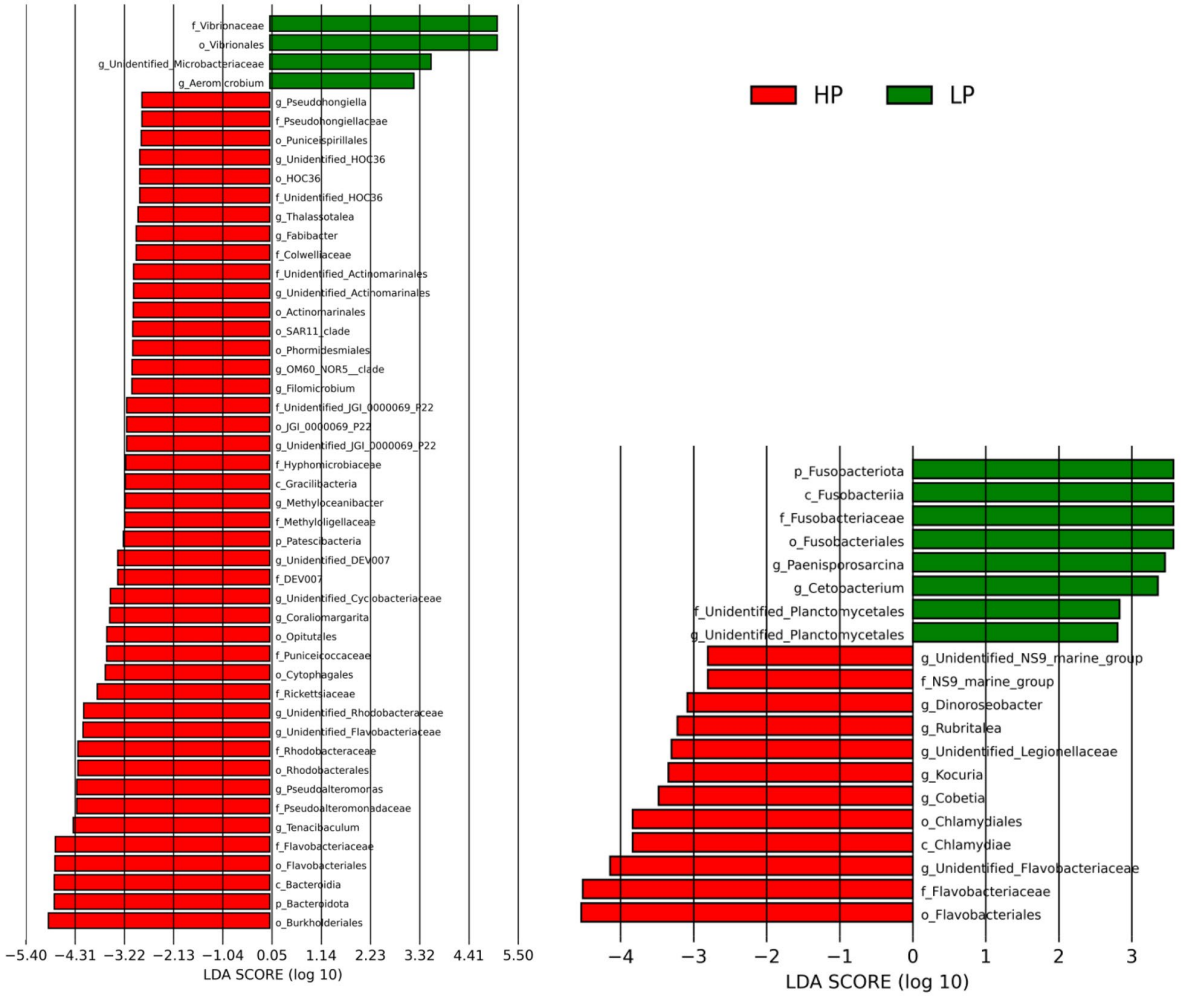
Most contributing taxa to differences between HP gill (red) and LP gill (green) bacterial communities. **Left, ***Diplodus annularis. ***Right, ***Pagellus acarne*. LDA scores were calculated using Linear discriminant analysis Effect Size (LEfSe), only bacterial taxa that raised an LDA score > 3 are shown

Then, we quantified how the relative abundance of bacterial taxa in gill mucus microbiota was related to parasite abundance. The abundance of all ASVs was aggregated to family or genus rank and then tested using Spearman’s rank correlation with the proportion of *Lamellodiscus* sp. found in the gills (parasitic load). The abundances of parasitic load of *Lamellodiscus spp.* displayed significant positive or negative correlations with the relative abundance of given bacterial taxa (Fig. 4). Significant differences in relative abundances of bacterial ASV among LP and HP fish were found (in both species and both tissues (linear discriminant analysis (LDA) effect size (LEfSe), Fig. 5).

Firstly, we observed that HP *D. annularis* gill microbiota was significantly more enriched in *Bacteroidota* (15.99% mean relative abundance (i.e., total sum scaling method) vs average of 2.32% in other LP fish) (LEfSe analysis, Fig. 5) and this proportion increased with parasite load in gill microbiota of both species (positive Spearman rank correlation, *p* = 0.001, ρ = 0.844 for *D. annularis* and *p* = 0.0132, ρ = 0.604 for *P. acarne*) (Fig. 4, Supplementary table S3). A medium positive correlation was found between the bacterial order *Flavobacteriales*, the bacterial family *Flavobacteriaceae* and the parasitic load of *Lamellodiscus* sp. in gills of *P. acarne* hosts (*p* = 0.021, ρ = 0.571 for both taxonomic rank) and a strong positive correlation in gills of *D. annularis* host (*p* = 0.001, ρ = 0.826; *p* = 0.003, ρ = 0.779) (Fig. 4, Supplementary table S3). In addition, this family was found in higher proportion in HP *D. annularis* gill microbiota (14.11% vs 1.28%) and HP *P. acarne* gill microbiota (9.99% vs 2.23%) (LEfSe analysis, Fig. 5).

The bacterial families *Rhodobactereaceae* and *Puniceicoccaceae* were positively correlated with the abundance of *Lamellodiscus sp.* in the gills of *D. annularis* (*p* = 0.001, ρ = 0.829 and *p* = 0.002, ρ = 0.803 respectively) as well as one unidentified *Rhodobactereaceae* genus in gill microbiota (*p* = 0.003, ρ = 0.769 respectively) of *D. annularis* (Fig. 4, Supplementary table S3).

In both tissues of *D. annularis*, a strong positive correlation was observed between parasitic load and the family *Pseudoalteromonadaceae* and more specifically with the genus *Pseudoalteromonas* (*p* = 0.001, ρ = 0.802 for both taxonomic rank in gill and *p* = 0.001, ρ = 0.811 for both taxonomic rank in skin) (Fig. 4, Supplementary table S3). Indeed, this bacterial family and genus were totally absent in gill microbiota of LP individuals (3.72% vs. 0% for both taxonomic rank) (LEfSe analysis, Fig. 5).

In contrast, the gill mucus microbiota of LP *D. annularis* contained significantly higher abundances of *Vibrionaceae* (32.9%), whereas this family was much less represented in other HP fish (9.95%) (LEfSe analysis, Fig. 5). These results were supported by strong negatives correlations between the abundance of *Lamellodiscus sp.* and the bacterial family *Vibrionaceae* or with the genus *Photobacterium* (*p* = 0.001, ρ=-0.890 and *p* = 0.028, ρ=-0.631 respectively) (Fig. 4, Supplementary table S3). Negative correlations were found with the genus *Enterovibrio* in gills (*p* = 0.016, ρ=-0.674) and skin (*p* = 0.038, ρ=-0.603) microbiota of *D. annularis* (Fig. 4, Supplementary table S3).

Another negative correlation was found between *Lamellodiscus* abundance and the *Micrococcales* order (*p* = 0.025, ρ =-0.638) and more specifically with the *Microbacteriacea* family (*p* = 0.011, ρ = -0.699) in *D. annularis* gills (Fig. 4, Supplementary table S3). In this family, the abundance of a non-identified genus is correlated with the parasitic load in both tissue of *D. annularis* (*p* = 0.007, ρ = -0.727 in gill and *p* = 0.05, ρ = -0.581 in skin) (Fig. 4, Supplementary table S3). This genus appears to be significantly more abundant in the gill microbiota of LP fish (LEfSe analysis, Fig. 5). The abundance of the genus *Blastopirellula* also decreased with parasitic load in *D. annularis* gill microbiota (*p* = 0.035, ρ = -0.609) (Fig. 4, Supplementary table S3).

In *P. acarne* individuals with low parasite load, gill microbiota appeared enriched with *Fusobacteria* (0.69% vs 0.05%), *Fusobacteriaceae* family (0.69% vs 0.05%) and *Propionigenium* genus, which is absent in HP gill microbiota (0.32% vs 0%) (LEfSe analysis, Fig. 5). A negative correlation was in particular observed for the family *Fusobacteriaceae* (Spearman’ rank order correlation, *p* = 0.048, ρ = -0.501) in *P. acarne* gill microbiota (Fig. 4, Supplementary table S3). A medium correlation between the parasitic load and the abundance of the family *Saprospiraceae* was found in the gills of *P. acarne* (*p* = 0.03, ρ = 0.54 and *p* = 0.002, ρ = 0.716 respectively) (Fig. 4, Supplementary table S3).

## Discussion

In this study, we examined the associations between gill and skin mucus bacterial communities of two wild sparid species, *D. annularis* and *P. acarne*, with contrasting parasitic loads of *Lamellodiscus* monogeneans.

Regarding general trends, *Proteobacteria* largely dominates the external microbiota of all individuals, which is consistent with previous studies [33–35]. If some bacteria could appear only transiently in the external mucus microbiota, our analysis with *D. annularis* samples without considering sequences retrieved from water samples indicated that the majority of bacterial communities are not reflections of the microbial assemblage of the surrounding water but result from selective mechanisms, such as suggested by previous studies [33, 34, 44, 48]. In consequence, even though these comparisons could not be done with *P. acarne* samples in the present study, it is likely that external mucus of these individuals harbor their own bacterial communities that differ from surrounding water. For one of the species we detected a significant effect of sampling date and location on the external microbiota, suggesting that the microbiota is variable and dynamic, shaped by environmental conditions. Gill and skin mucus also contain tissue-specific and species-specific assemblages. These results are consistent with previous studies that shown that both environmental [49, 50] and host-associated factors shape fish microbiota [29, 34, 48, 51].

We identified differences in the diversity of the microbiota between LP and HP of *P. acarne*, with the HP mucus appearing to be richer and more diverse than the LP mucus. In *D. annularis*, we did not identified any differences in the diversity of the microbiota between HP and LP gill or skin mucus. However, we identified a positive correlation between parasitic load and microbiota diversity in skin mucus. This is not in line with the results of [35] who found that an increase of parasite diversity is linked to a decrease of gill microbiota diversity. Nevertheless, other studies highlighted that an increase in alpha diversity indices follows parasitic infestation. Llewellyn and coll. [52] showed that the experimental infection of Atlantic salmon with salmon lice resulted in an increased diversity of the skin microbiota in infected fish. Zhang and coll. [53] showed that a group of rainbow trout experimentally parasitized by *Ichthyophthirius multifiliis* presented higher values for alpha diversity in skin microbiota than a control group. Overall, these results suggest that the relation between parasites and diversity of the external microbiota greatly varies depending on the host and parasite species.

The effect of the sampling date on parasitic load is not the same depending on the host species considered. In the case of *D. annularis*, sampling date had a significant effect on the parasite load in *Lamellodiscus*, even though the sampling period was limited in time (2 summer months). In the case of *P. acarne*, sampling was carried out over two months in different seasons, but without any effect on the parasite load in *Lamellodiscus.* These results suggest that several factors are involved in the occurrence and abundance of monogeneans in fish, such as environmental factors like salinity [54] or water temperature [55, 56]. However, the effect of water temperature does not seem to be the same for all monogenean species. Some authors have shown that some *Dactylogyrus* species seem to prefer warm temperatures (*D. vastator*, *D. ctenopharyngodonis*) while others seem to prefer colder temperatures (*D. lamellatus*, *D. extensus*) [57, 58].

Using the analysis of bacterial communities, we identified bacterial families and genera that correlated positively or negatively with the parasite load in *Lamellodiscus*. We found a strong correlation between the abundance of *Flavobacteriaceae* and the parasite load in the gill mucus of both species. Similar results have already been reported: this family of bacteria is often found after a parasitic infection. For example, Zhang and coll. [53] showed that experimental infection with *Ichthyophthirius multifiliis* (ciliate) reduced the abundance of skin commensals and increased the intensity of *Flavobacteriaceae* in rainbow trout skin. Similarly, an in vivo study showed that Atlantic salmon infected with sea lice, the ectoparasitic copepod *Lepeophtheirus salmonis*, were susceptible to skin colonization by known pathogenic bacteria genera, belonging in particular to the *Flavobacteriaceae* family [52]. Among these bacterial genera was *Tenacibaculum sp.*, a genus that we found correlated with the parasitic load in the gills of *D. annularis*. Unfortunately, we could not identify the nature of all the bacteria belonging to the *Flavobacteriaceae* family from our dataset as most remain unidentified at the species or even the genus level. As a result, it remains difficult to speculate on the pathogenicity of these bacteria associated with fish with high parasitic loads. However, numerous studies have shown that fish infected with ectoparasites [59, 60] and especially with monogeneans are more susceptible to numerous pathogenic bacteria [61, 62]. It is therefore likely that the increase in *Flavobacteriaceae* abundance with the parasitic load observed in our samples involves potentially pathogenic bacteria belonging in particular to this family. Several hypotheses have been proposed to explain the increased susceptibility of parasitized fish to bacterial infections. Firstly, the artificial abrasion created by the attachment and feeding mode of parasite can induce histopathological changes in host tissues, which are likely to create a gateway for secondary infections [22, 53, 63, 64]. Thus, skin and gill injuries caused by monogeneans would increase the adhesion of pathogenic bacteria. It is also possible that primary infection by a pathogen induces immune stress in the host, reducing its resistance to opportunistic bacterial infections [65]. This could also be the result of the modulation of the immune system by the parasite. Several studies have shown that a variety of strategies can be implemented to circumvent and/or reduce the host immune response, leading to reduced resistance to secondary bacterial infections [66–70]. It has been shown for the monogenean *Eudiplozoon nipponicum*’s excretory-secretory products (cysteine peptidase inhibitors) lead to a down-regulation of two cytokines produced by macrophages which may prevent inflammation at the invasion site and increased susceptibility to concomitant bacterial infections [71]. Another example of immunomodulation was identified during an infestation of the gilthead sea bream (*Sparus aurata*) by the monogenean *Sparicotyle chrysophrii*. The parasite was found to inhibit the humoral immune system, slowing down the assembly process of its host’s complement complex [72] and a splenic down-regulation of genes implicated in inflammation or apoptosis [73], which may result in a delayed response to secondary infections. Finally, an experimental study showed that a primary infection of *Carassius auratus* by *Dactylogyrus intermedius* induced the down-regulation of two immune-related factors (TGF beta and C3). The same study also found that parasitized fish exhibited significantly higher loads of *Flavobacterium columnare*, suggesting that parasitic infection can enhance bacterial invasion [64]. Taken together, these studies suggest that the effects of *Lamellodiscus* monogeneans on the epithelium and host immunity can be responsible for the observed changes in bacterial communities and the increase in potentially pathogenic bacterial taxa. However, other bacterial families and genera that were positively correlated with the parasite load in *Lamellodiscus* monogeneans could not be attributed to any known pathogen and their association with parasitism remains difficult to determine. One hypothesis is that these bacteria could attract parasites in particular through their metabolites. For example, an in vitro experiment with *Strongyloides* nematodes showed that several isolated bacteria act as key chemosensory cues to guide parasite movement but further studies are needed to characterize the metabolites involved [74].

The origin of the bacteria linked to parasitic infection remains unclear; they could be opportunistic bacteria naturally present in the marine environment or belong to the parasite’s microbiota. Parasite could act as a vector for bacteria, carrying them directly to the host. A recent study showed that the microbiota of infected *Leuciscus burdigalensis* fins was highly similar to that of the adult and larval crustacean ectoparasite *Tracheliastes polycolpus* [18]. The authors suggested that the bacteria shared between the infected fin and the crustacean ectoparasite result from a co-infection dynamic between the parasite and its associated microbiota. It has also been shown that the parasite *Ichthyophthirius multifiliis* can transmit the bacterial pathogen *Edwardsiella ictaluri* to channel catfish [75]. To our knowledge, microbiota associated with monogeneans is not yet known, although the presence of bacteria on their surfaces has long been observed [76]. Several studies on ectoparasitic copepods have reported the presence of *Flavobacteriaceae* in their microbiota. For example, a study in 2020 showed that the microbiota of the Atlantic salmon ectoparasite *Lepeophtheirus salmonis* was dominated by the order *Flavobacteriales* [77]. Similar results were found in the microbiota of the copepod *Caligus rogercresseyi*, which is also known to parasitise the Atlantic Salmon [78]. Interestingly, these authors identified several taxa with the ability to secrete bioactive compounds, such as the genus *Pseudoaltermonas*, on the copepod microbiota. In the present study, we found that this bacterial genus was positively correlated with parasite load in the skin and gills of *D. annularis*. These microorganisms are known to secrete bioactive compounds with antialgae and antimicrobial properties [79]. A possible role of this bacterial genus could be to increase their resistance and persistence in the fish host. However, it is difficult to determine their exact role in the infestation process as all the bacterial species present in our samples could not be identified. Whether the change in microbiota between parasitized and non-parasitized hosts is due to opportunistic bacteria in the environment or to the contribution of the parasite’s microbiota, it is crucial to understand the dynamics of co-infection between bacterial pathogens and monogeneans. Indeed, these co-infections are often associated with greater damage than that resulting from the isolated presence of just one of either pathogen alone [61, 64, 80, 81].

Interestingly, we found that *Vibrionaceae* were significantly enriched and negatively correlated to the parasitic load in gills of LP *D. annularis* fish and the same results were obtained with *Fusobacteria* in gills of LP *P. acarne*. Similar result was already reported in 2020, who found higher abundance of *Fusobacteria* and *Vibrionaceae* in gill mucus of unparasitized butterflyfish *Chaetodon lunalatus* compared to other *Chaetodon* species parasitized by *Haliotrema* monogeneans [28]. The authors proposed that *Fusobacteria* increase the mucus production in fish, possibly leading to a thicker layer of mucus on their gills. This thickening could lead to lower oxygen levels, potentially promoting higher hemoglobin levels. Furthermore, a positive correlation was identified between *Fusobacteria* and three hemoglobin-derived peptides, which may act as antimicrobial and antiparasitic agents and could be produced by extracellular protease microbes that specifically cleave hemoglobin. Similarly, it has been shown that the bogue (*Boops boops*), a sparid species never parasitized by *Lamellodiscus* monogeneans, had a higher abundance of *Fusobacteria* and *Vibrionaceae* in their gill mucus microbiota than parasitized sparids [35]. Similarly, a study of three tropical fish species - *Epinephelus fuscoguttatus*, *Epinephelus sexfasciatus* and *Atule mate -* have reported negative correlations between intestinal endoparasites (such as digeneans, nematode or cestodes) and the abundance of *Vibrio* and *Photobacterium* [82]. We also found a strong negative correlation between the *Photobacterium* genus and the parasitic load in *D. annularis* gill microbiota. Interestingly, particularly high relative abundances of *Photobacterium* spp. were found in the only four individuals without *Lamellodiscus* in their gills.

To date, *Vibrionaceae* have primarily been investigated due to their pathogenic potential to humans and aquatic animals but some members of this family are known to display antibacterial effects via secondary metabolites [83, 84]. Little is known on their effect on eukaryotic organisms such as monogeneans. In a 2014 study, a lethal effect of two *Vibrionaceae* species, *Photobacterium halotolerans* and *Vibrio coralliilyticus*, have been observed on two eukaryotic species (*Artemia* sp. and *Caernorhabitis elegans*) [85]. However, the secondary metabolites or specific mechanisms involved in this toxicity could not be determined. Finally, *Vibrionaceae* have already been described as being able to inhibit the settlement and attachment of cyprids in macroalgae, but the mechanisms involved have not yet been deciphered [86]. These studies suggest that members of the *Vibrionaceae* family are capable of secreting potentially host-protective bioactive compounds. Future studies are needed to test the effect of these bioactive compounds on the host specificity of monogeneans.

## Conclusion

In conclusion, our findings highlight the dynamic nature of fish microbiota in response to parasitic infestations, emphasizing the need for a comprehensive understanding of co-infections dynamics. The observed correlations between specific bacterial families or genera and parasitic load highlight the complex interplay between host, parasite and bacterial communities. Future investigations should explore the specific mechanisms underlying these interactions and the potential applications of bacteria in mitigating parasitic infections in fish.

### Electronic supplementary material

Below is the link to the electronic supplementary material.

Supplementary Material 1

## Data Availability

Sequence data will be available upon publication in the NCBI Sequence Read Archive (SRA, https://www.ncbi.nlm.nih.gov/sra) database under the BioProject PRJNA1050994 (https://dataview.ncbi.nlm.nih.gov/object/PRJNA1050994?reviewer=1au0f72tav24kfm8qkk40jktbq).

## Abbreviations

ASV: Amplicon Sequence Variant
LP: Lightly Parasitized
HP: Heavily Parasitized
ANOVA: Analysis Of Variance
KW: Kruskal-Wallis
CI: Conover-Iman
PCoA: Principal Coordinates Analysis
LEfSe: Linear Discriminant Analysis Effect Size
LDA: Linear Discriminant Analysis
PERMANOVA: Permutational Multivariate Analysis Of Variance
BC: Bray-Curtis Dissimilarity Index
WU: Weighted Unifrac Distance
