# Supplementary material for: Link between bacterial communities and contrasted loads in ectoparasitic monogeneans from the external mucus of two wild sparid species (Teleostei)
Source: Anim Microbiome. 2024 Jul 30;6:42. doi: 10.1186/s42523-024-00329-0 (PMC11290237; doi:10.1186/s42523-024-00329-0)
Supplement: Supplementary file 1 — Supplementary Material 1 [file 42523_2024_329_MOESM1_ESM.docx]

**Table S1:** Place of capture, collection date and *Lamellodiscus* load of each fish sampled for this study. HP, Heavily Parasitized; LP, Lightly Parasitized

| **Fish** | **Place of capture** | **Date** | ***Lamellodiscus* load** | **Parasitism** |
| --- | --- | --- | --- | --- |
| *Diplodus annularis* 1 | Banyuls-sur-Mer | July 2021 | 90 | HP |
| *Diplodus annularis* 2 | Banyuls-sur-Mer | July 2021 | 49 | HP |
| *Diplodus annularis* 3 | Banyuls-sur-Mer | July 2021 | 26 | HP |
| *Diplodus annularis* 4 | Banyuls-sur-Mer | June 2021 | 23 | HP |
| *Diplodus annularis* 5 | Banyuls-sur-Mer | July 2021 | 20 | HP |
| *Diplodus annularis* 6 | Banyuls-sur-Mer | June 2021 | 17 | HP |
| *Diplodus annularis* 7 | Banyuls-sur-Mer | June 2021 | 8 | LP |
| *Diplodus annularis* 8 | Banyuls-sur-Mer | June 2021 | 6 | LP |
| *Diplodus annularis* 9 | Banyuls-sur-Mer | June 2021 | 0 | LP |
| *Diplodus annularis* 10 | Banyuls-sur-Mer | June 2021 | 0 | LP |
| *Diplodus annularis* 11 | Banyuls-sur-Mer | June 2021 | 0 | LP |
| *Diplodus annularis* 12 | Banyuls-sur-Mer | June 2021 | 0 | LP |
| *Pagellus acarne* 1 | Banyuls-sur-Mer | July 2021 | 189 | HP |
| *Pagellus acarne* 2 | Argelès-sur-Mer | March 2022 | 136 | HP |
| *Pagellus acarne* 3 | Argelès-sur-Mer | July 2021 | 84 | HP |
| *Pagellus acarne* 4 | Argelès-sur-Mer | March 2022 | 82 | HP |
| *Pagellus acarne* 5 | Argelès-sur-Mer | July 2021 | 64 | HP |
| *Pagellus acarne* 6 | Argelès-sur-Mer | July 2021 | 58 | HP |
| *Pagellus acarne* 7 | Argelès-sur-Mer | July 2021 | 32 | HP |
| *Pagellus acarne* 8 | Argelès-sur-Mer | March 2022 | 23 | HP |
| *Pagellus acarne* 9 | Argelès-sur-Mer | March 2022 | 19 | LP |
| *Pagellus acarne* 10 | Banyuls-sur-Mer | July 2021 | 19 | LP |
| *Pagellus acarne* 11 | Banyuls-sur-Mer | March 2022 | 12 | LP |
| *Pagellus acarne* 12 | Banyuls-sur-Mer | June 2021 | 7 | LP |
| *Pagellus acarne* 13 | Argelès-sur-Mer | July 2021 | 4 | LP |
| *Pagellus acarne* 14 | Banyuls-sur-Mer | July 2021 | 1 | LP |
| *Pagellus acarne* 15 | Banyuls-sur-Mer | July 2021 | 0 | LP |
| *Pagellus acarne* 16 | Argelès-sur-Mer | July 2021 | 0 | LP |
| Water 1 | Banyuls-sur-Mer | June 2021 | - | - |
| Water 2 | Banyuls-sur-Mer | June 2021 | - | - |
| Water 3 | Banyuls-sur-Mer | July 2021 | - | - |
| Water 4 | Banyuls-sur-Mer | March 2022 | - | - |

**Figure S1:** Comparison of alpha diversity values between tissue of *D. annularis* or *P. acarne* and water. Faith’s phylogenetic diversity and Shannon diversity index for skin mucus (grey), gill mucus (brown) and water communities (blue) Significant (a) and non‐significant (b) differences between tissue are indicated (for Faith index: post hoc Tukey test significant if p-value<0.05. For Shannon index: Post hoc Conover-Iman test significant if *p* < alpha/2 = 0.025)

**Table S2:** Results of the LM and GLM analysis for bacterial diversity for each species. Significant p-value are in bold

|  | **p-value** |  |
| --- | --- | --- |
|  | **Faith** | **Shannon** |
| ***Diplodus annularis*** |  |  |
| Tissue | 0.07 | **0.02** |
| HP vs LP | 0.07 | 0.11 |
| Collection date | **<0.001** | 0.14 |
| Tissue x HPLP | 0.73 | 0.58 |
| Tissue x Collection date | 0.47 | 0.89 |
|  |  |  |
|  |  |  |
| ***Pagellus acarne*** |  |  |
| Tissue | **0.003** | **<0.001** |
| HP vs LP | **0.02** | 0.57 |
| Collection date | 0.43 | 0.87 |
| Place of capture | 0.07 | **0.02** |
| Tissue x HPLP | 0.55 | 0.30 |
| Tissue x Collection date | 0.61 | 0.46 |
| Tissue x Place of capture | **0.004** | **0.04** |
| HP vs LP x Collection date | 0.40 | 0.60 |
| HP vs LP x Place of capture | 0.15 | 0.18 |

**Figure S2:** PCoA plot based on dissimilarities between bacterial communities of Diplodus annularis using Bray Curtis (top) and weighted Unifrac (bottom). Form indicates the type of tissue, gill mucus (circle), skin mucus (triangle) and color refers to lightly parasitized (LP, green) or heavily parasitized (HP, red).

**Figure S3:** Significant correlation between skin microbiota diversity (Faith’s phylogenetic index) and *Lamellodiscus* load in *D. annularis* (Pearson correlation test, p<0.05, R=0.885).

**Table S3:** Significant Spearman correlation coefficients between the abundances of *Lamellodiscus* and bacterial phylum, class, order, family and genus in *Diplodus annularis* and *Pagellus acarne* gills mucus

| ***Diplodus annularis* gill** |  |  |  | ***Pagellus acarne* gill** |  |  |
| --- | --- | --- | --- | --- | --- | --- |
|  | **P-value** | **Coef** |  |  | **P-value** | **Coef** |
| **Phylum** |  |  |  | **Phylum** |  |  |
| Patescibacteria | 0.05 | 0.576 |  | Fusobacteriota | 0.048 | -0.501 |
| Bacteroidota | <0.001 | 0.844 |  | Bacteroidota | 0.013 | 0.604 |
|  |  |  |  |  |  |  |
| **Class** |  |  |  | **Class** |  |  |
| Gracilibacteria | 0.007 | 0.733 |  | Fusobacteriia | 0.048 | -0.501 |
| KD4-96 | 0.006 | 0.738 |  | Chlamydiae | 0.022 | 0.569 |
| Rhodothermia | 0.005 | 0.748 |  | Bacteroidia | 0.013 | 0.604 |
| Bacteroidia | <0.001 | 0.844 |  |  |  |  |
|  |  |  |  | **Order** |  |  |
| **Order** |  |  |  | Fusobacteriales | 0.048 | -0.501 |
| Vibrionales | <0.001 | -0.89 |  | Defluviicoccales | 0.045 | 0.507 |
| Corynebacteriales | 0.02 | -0.659 |  | Chitinophagales | 0.031 | 0.54 |
| Micrococcales | 0.026 | -0.638 |  | Chlamydiales | 0.022 | 0.569 |
| Unidentified Gammaproteobacteria | 0.043 | 0.59 |  | Flavobacteriales | 0.021 | 0.571 |
| Cytophagales | 0.043 | 0.591 |  |  |  |  |
| Rhodothermales | 0.038 | 0.602 |  | **Family** |  |  |
| Mycoplasmatales | 0.038 | 0.602 |  | Unidentified Planctomycetales | 0.047 | -0.503 |
| Cardiobacteriales | 0.038 | 0.602 |  | Fusobacteriaceae | 0.048 | -0.501 |
| Micavibrionales | 0.036 | 0.607 |  | Unidentified Defluviicoccales | 0.042 | 0.514 |
| Oceanospirillales | 0.018 | 0.667 |  | Saprospiraceae | 0.031 | 0.54 |
| JGI 0000069-P22 | 0.007 | 0.733 |  | Flavobacteriaceae | 0.021 | 0.571 |
| Unidentified KD4-96 | 0.006 | 0.738 |  | Spongiibacteraceae | 0.02 | 0.575 |
| Actinomarinales | 0.004 | 0.757 |  | NS9 marine group | 0.015 | 0.594 |
| Phormidesmiales | 0.002 | 0.792 |  |  |  |  |
| Flavobacteriales | 0.001 | 0.826 |  | **Genus** |  |  |
| Opitutales | 0.001 | 0.828 |  | Propionigenium | 0.027 | -0.551 |
| Rhodobacterales | 0.001 | 0.829 |  | Unidentified Planctomycetales | 0.047 | -0.503 |
|  |  |  |  | Rubidimonas | 0.049 | 0.499 |
| **Family** |  |  |  | Unidentified Defluviicoccales | 0.042 | 0.514 |
| Vibrionaceae | <0.001 | -0.89 |  | Aquibacter | 0.023 | 0.563 |
| Microbacteriaceae | 0.011 | -0.699 |  | Fabibacter | 0.021 | 0.571 |
| Unidentified Microtrichales | 0.042 | -0.594 |  | Unidentified NS9 marine group | 0.015 | 0.594 |
| Fokiniaceae | 0.048 | 0.58 |  | NS4 marine group | 0.008 | 0.64 |
| Parvularculaceae | 0.048 | 0.58 |  | Unidentified Flavobacteriaceae | 0.001 | 0.739 |
| Unidentified Gammaproteobacteria | 0.043 | 0.59 |  |  |  |  |
| Hyphomonadaceae | 0.043 | 0.591 |  |  |  |  |
| Rhodothermaceae | 0.038 | 0.602 |  |  |  |  |
| Mycoplasmataceae | 0.038 | 0.602 |  |  |  |  |
| Cardiobacteriaceae | 0.038 | 0.602 |  |  |  |  |
| Methylophilaceae | 0.026 | 0.635 |  |  |  |  |
| Unidentified JGI 0000069-P22 | 0.007 | 0.733 |  |  |  |  |
| Unidentified KD4.96 | 0.006 | 0.738 |  |  |  |  |
| Colwelliaceae | 0.006 | 0.741 |  |  |  |  |
| Phormidesmiaceae | 0.005 | 0.748 |  |  |  |  |
| Clade II | 0.005 | 0.748 |  |  |  |  |
| Unidentified Actinomarinales | 0.004 | 0.757 |  |  |  |  |
| Pseudohongiellaceae | 0.004 | 0.758 |  |  |  |  |
| Flavobacteriaceae | 0.003 | 0.779 |  |  |  |  |
| Rickettsiaceae | 0.002 | 0.8 |  |  |  |  |
| Pseudoalteromonadaceae | 0.002 | 0.802 |  |  |  |  |
| Puniceicoccaceae | 0.002 | 0.803 |  |  |  |  |
| Rhodobacteraceae | 0.001 | 0.829 |  |  |  |  |
|  |  |  |  |  |  |  |
| **Genus** |  |  |  |  |  |  |
| Unidentified Microbacteriaceae | 0.007 | -0.727 |  |  |  |  |
| Enterovibrio | 0.016 | -0.674 |  |  |  |  |
| Photobacterium | 0.028 | -0.631 |  |  |  |  |
| Aeromicrobium | 0.035 | -0.611 |  |  |  |  |
| Blastopirellula | 0.035 | -0.609 |  |  |  |  |
| Unidentified Microtrichales | 0.042 | -0.594 |  |  |  |  |
| Cyanobium PCC.6307 | 0.048 | 0.58 |  |  |  |  |
| MD3.55 | 0.048 | 0.58 |  |  |  |  |
| Parvularcula | 0.048 | 0.58 |  |  |  |  |
| Unidentified Gammaproteobacteria | 0.043 | 0.59 |  |  |  |  |
| Unidentified Rhodothermaceae | 0.038 | 0.602 |  |  |  |  |
| Mycoplasma | 0.038 | 0.602 |  |  |  |  |
| Unidentified Cardiobacteriaceae | 0.038 | 0.602 |  |  |  |  |
| Unidentified Cyclobacteriaceae | 0.029 | 0.627 |  |  |  |  |
| Methylotenera | 0.026 | 0.635 |  |  |  |  |
| Phormidesmis ANT.LACV5.1 | 0.022 | 0.651 |  |  |  |  |
| Unidentified Rickettsiaceae | 0.021 | 0.654 |  |  |  |  |
| Sphingorhabdus | 0.021 | 0.654 |  |  |  |  |
| Marinicella | 0.019 | 0.662 |  |  |  |  |
| Tenacibaculum | 0.013 | 0.691 |  |  |  |  |
| Fabibacter | 0.008 | 0.724 |  |  |  |  |
| Unidentified JGI 0000069-P22 | 0.007 | 0.733 |  |  |  |  |
| Unidentified KD4-96 | 0.006 | 0.738 |  |  |  |  |
| Acrophormium PCC.7375 | 0.005 | 0.748 |  |  |  |  |
| Unidentified Clade II | 0.005 | 0.748 |  |  |  |  |
| Unidentified Actinomarinales | 0.004 | 0.757 |  |  |  |  |
| Pseudohongiella | 0.004 | 0.758 |  |  |  |  |
| Thalassotalea | 0.004 | 0.758 |  |  |  |  |
| Unidentified Flavobacteriaceae | 0.004 | 0.767 |  |  |  |  |
| Unidentified Rhodobacteraceae | 0.003 | 0.769 |  |  |  |  |
| Megaira | 0.003 | 0.776 |  |  |  |  |
| Coraliomargarita | 0.002 | 0.792 |  |  |  |  |
| Pseudoalteromonas | 0.002 | 0.802 |  |  |  |  |
